# Comparative structural, kinetic and inhibitor studies of *Trypanosoma brucei* trypanothione reductase with *T. cruzi*^☆^

**DOI:** 10.1016/j.molbiopara.2009.09.002

**Published:** 2010-01

**Authors:** Deuan C. Jones, Antonio Ariza, Wing-Huen A. Chow, Sandra L. Oza, Alan H. Fairlamb

**Affiliations:** a College of Life Sciences, The Wellcome Trust Biocentre, University of Dundee, Dundee DD1 5EH, Scotland, UK; b Ecole Supérieure de Biotechnologie Strasbourg, Parc d’Innovation, Boulevard Sébastien Brandt, 67412 Illkirch, France

**Keywords:** TryR, trypanothione reductase, T(S)_2_, trypanothione disulphide, DTNB, 5,5′-dithio-bis(2-nitrobenzoic acid), HAT, human African trypanosomiasis, Trypanothione metabolism, Trypanosome, Thiol, Enzymology, Drug discovery

## Abstract

As part of a drug discovery programme to discover new treatments for human African trypanosomiasis, recombinant trypanothione reductase from *Trypanosoma brucei* has been expressed, purified and characterized. The crystal structure was solved by molecular replacement to a resolution of 2.3 Å and found to be nearly identical to the *T. cruzi* enzyme (root mean square deviation 0.6 Å over 482 Cα atoms). Kinetically, the *K*_m_ for trypanothione disulphide for the *T. brucei* enzyme was 4.4-fold lower than for *T. cruzi* measured by either direct (NADPH oxidation) or DTNB-coupled assay. The *K*_m_ for NADPH for the *T. brucei* enzyme was found to be 0.77 μM using an NADPH-regenerating system coupled to reduction of DTNB. Both enzymes were assayed for inhibition at their respective S = *K*_m_ values for trypanothione disulphide using a range of chemotypes, including CNS-active drugs such as clomipramine, trifluoperazine, thioridazine and citalopram. The relative IC_50_ values for the two enzymes were found to vary by no more than 3-fold. Thus trypanothione reductases from these species are highly similar in all aspects, indicating that they may be used interchangeably for structure-based inhibitor design and high-throughput screening.

## 1 Introduction

**Fig. 1:**
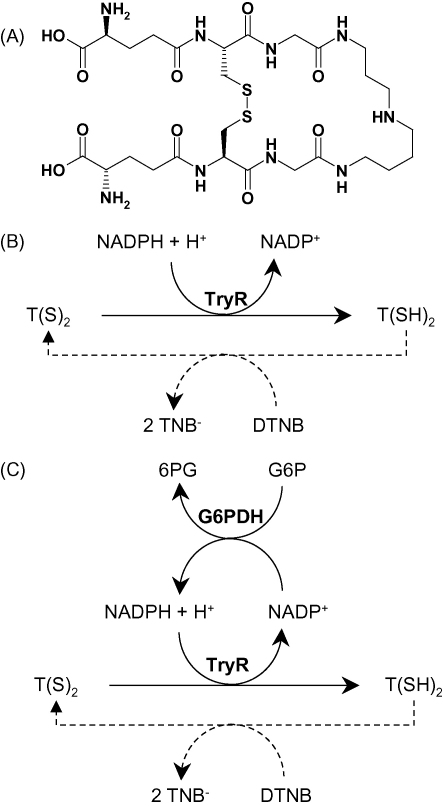
Trypanothione reductase assay principle. Panel A, structure of trypanothione disulphide. Panel B, in the DTNB-coupled assay trypanothione is recycled to trypanothione disulphide by the reduction of DTNB. Formation of thionitrobenzoate anion (TNB^-^) is monitored at 412 nm. Panel C, in a modification of the DTNB-coupled assay NADPH is recycled by the enzyme glucose-6-phosphate dehydrogenase.

*Trypanosoma brucei* is a parasitic protozoan of the family Trypanosomatidae (order Kinetoplastida, suborder Trypanosomatina) responsible for human African trypanosomiasis, also called sleeping sickness. The East and West African forms of the disease are caused by the *T. b. rhodesiense* and *T. b. gambiense* subspecies, respectively [1]. The disease is fatal if untreated, and the few available drugs are not ideal due to emerging drug resistance; parenteral administration; toxic side-effects and cost [2]. *T. b. brucei*, one of the causative agents of Nagana cattle disease, can serve as a model organism for drug discovery and is non-pathogenic to humans. *T. brucei* subspecies, along with all parasites of the order Kinetoplastida, possesses a novel thiol called trypanothione [*N*^1^, *N*^8^-bis(glutathionyl)spermidine] [3]. One of the major roles of this metabolite is to protect the parasite from oxidative stress by maintaining a reducing environment in the cell. In most other organisms, in particular mammals, it is glutathione that plays this protective role. Protection of the parasite against oxidative stress is achieved through the oxidation of the dithiol form of trypanothione (T(SH)_2_) into the disulphide form (T(S)_2_), followed by regeneration of T(SH)_2_ by the NADPH-dependent enzyme trypanothione reductase (TryR) (Fig. 1) [4]. A similar mechanism involving glutathione and glutathione reductase is observed in other organisms, including humans. However, the enzymes trypanothione reductase and glutathione reductase are highly specific for their respective disulphide substrates [5] such that selective inhibition by small molecules can be readily achieved [6].

Metabolism of trypanothione and other low molecular weight thiols has been established as an attractive target for drug discovery in several trypanosomatids [7–9] and TryR from *T. b. brucei* has been specifically validated as a drug target, *inter alia*, by conditional knockout experiments [10]. However, kinetic and inhibition studies of the *T. b. brucei* enzyme have not been developed. Previously the *T. cruzi* enzyme has been used to guide drug discovery for human African trypanosomiasis (HAT), but absence of a clear correlation between inhibitor potency against *T. cruzi* TryR and cidal activity against bloodstream forms of *T. b. brucei* has raised concerns that the *T. cruzi* enzyme is not a suitable model for the *T. b. brucei* enzyme [6]. To address this issue, we report here a comprehensive comparative study of the physicochemical properties, structure, kinetics and inhibitor sensitivities of these enzymes. The information on the enzyme from *T. b. brucei* is also of particular relevance since it is identical at the amino acid level to the putative TryR from *T. b. gambiense*, the causative agent of over 90% of reported HAT cases [11].

## 2 Materials and methods

### 2.1 Organisms and reagents

Routine plasmid manipulations were performed in *Escherichia coli* strain JM109 and over-expression in strain BL21 Star (DE3)pLysS (Invitrogen). All chemicals were of the highest grade available from Sigma, BDH and Molecular Probes. Restriction enzymes and DNA-modifying enzymes were from Promega or Roche.

### 2.2 Cloning and expression TbTryR in *E. coli*

The complete open reading frame of *TbTRYR* was amplified by PCR from genomic DNA from *T. b. brucei* strain S427 (MITat 1.4) using primers based on a putative TryR gene sequence deposited in GeneDB (**Tb10.406.0520**). The primers used for amplification were: 5′-CAT **ATG** TCC AAG GCC TTC GAT TTG G-3′ and 5′-GGA TCC **TTA** CAG GTT AGA GTC CGG AAG C-3′, incorporating the NdeI and BamHI restriction sites (underlined), respectively, with the start and stop codons in bold.

PCR amplification was done in triplicate. After sequencing, the PCR product of ∼1.49 kb was then cloned (via a TOPO cloning vector) into the NdeI/BamHI site of pET3a to generate plasmid pET3a-*Tb*TryR. A 4 L culture of BL21 Star (DE3)pLysS/pET3a-*Tb*TryR was grown to test expression and purification. The cells were grown at 37 °C in LB media, containing 50 μg ml^1^ carbenicillin for selection of pET3a and 12.5 μg ml^1^ chloramphenicol for the selection of pLysS, at 37 °C with moderate agitation (200 rpm). A larger scale expression in a 30 L culture was grown in a fermenter (Infors HT) using the same media and antibiotics at 37 °C. When the cultures reached an *A*_600_ of ∼0.6, isopropyl-β-d-thiogalactopyranoside was added to a final concentration of 0.5 mM. Cultures were grown for an additional 16 h and then harvested by centrifugation at 3480 × *g* at 4 °C for 30 min and washed in phosphate buffered saline (137 mM NaCl, 2.68 mM KCl, 10.1 mM Na_2_HPO_4_, 1.76 mM KH_2_PO_4_).

### 2.3 Purification of TbTryR

*E. coli* cells were lysed using a one-shot cell disruptor (Constant Systems Ltd.). Purification of recombinant *Tb*TryR was achieved by a combination of ammonium sulphate purification, affinity chromatography on 2′5′-ADP Sepharose, and anion exchange chromatography essentially as described previously [12]. Purity was assessed by SDS-PAGE.

*Tb*TryR was used directly from this procedure for crystallography, analysis of flavin content and measurement of extinction coefficient. The remainder of the TryR was precipitated with 70% saturating ammonium sulphate and aliquotted for storage at 4 °C for subsequent use in kinetic experiments. Protein concentration was measured using the method of Bradford with bovine serum albumin as a standard [13].

### 2.4 Assessment of oligomeric state

*Tb*TryR (600 μg) was applied to a gel filtration column (Superdex 200 10/300 GE Healthcare) previously equilibrated with 25 mM HEPES pH 7.5 containing 100 mM NaCl. Elution of the column was monitored at 280 nm using an Akta purifier. Molecular weight was inferred from comparison with standards (BioRad gel filtration standard) on a plot of elution volume versus Log molecular weight. Samples of the recombinant enzyme were also analysed by analytical ultracentrifugation (Analytical ultracentrifugation service, College of Life Sciences, University of Dundee).

### 2.5 Absorbance spectra and determination of absorption coefficient

All spectra were carried out in a UV-1601pc temperature-regulated spectrophotometer (Shimadzu) using 1-cm path-length quartz cuvettes (200 μl sample volume). Enzymes were extensively dialysed against 40 mM HEPES pH 7.4, 1 mM EDTA. Absorbance spectra were acquired over a range of 200–800 nm. The enzyme-associated flavin was liberated by thermal denaturation at 100 °C for 20 min in the presence of 10 mM MgCl_2_. Denatured protein was removed by microcentrifugation and the concentration of free flavin determined from its absorption coefficient at 450 nm (11.3 mM^−1^ cm^−1^). The absorption coefficient of oxidised *Tb*TryR was calculated from the absorbance at 463 nm/[FAD] in triplicate samples. The absorption coefficient of NADPH-reduced *Tb*TryR at 530 nM was calculated from the absorbance at 530 nm/[FAD].

### 2.6 Enzyme assays

TryR was assayed spectrophotometrically either by monitoring the trypanothione-dependent oxidation of NADPH at 340 nm [14], or by the reduction of 5,5′-dithio-bis(2-nitrobenzoic acid) (DTNB) at 412 nm (see Fig. 1B) [15]. Assays at 340 nm were carried out at 25 °C in 500 μl volume acrylic cuvettes and changes in absorbance monitored with a UV-1601 PC spectrophotometer (Shimadzu). The standard assay mixture contained 40 mM HEPES pH 7.4, 1 mM EDTA, ∼10 mU ml^−1^ TryR, 150 μM NADPH and 100 μM T(S)_2_. Assays at 412 nm were carried out in 96-well plates (Polysorp, Nunc) at room temperature in a volume of 200 μl. Changes in absorbance were monitored in a SpectraMax 340pc plate reader (Molecular Devices). The standard assay mixture contained 40 mM HEPES pH 7.4, 1 mM EDTA, ∼10 mU ml^−1^ TryR, 150 μM NADPH, 6 μM T(S)_2_ and 50 μM DTNB. All assays were initiated by addition of the T(S)_2_. Data was collected using SoftMax Pro (molecular devices) and UVProbe 2 (Shimadzu) software, processed and analysed using Excel 2002 (Microsoft) and GraFit 5 (Erithacus Software). NADPH stock solutions were measured spectrophotometrically at 340 nm using an absorption coefficient of 6.22 × 10^3^ M^−1^ cm^−1^[16]. The concentration of T(S)_2_ stock solutions were determined by measuring the oxidation of NADPH in the presence of excess TryR.

### 2.7 *K*_m_ analyses

*K*_m_ values with respect to T(S)_2_ were determined for *Tb*TryR in both assays using purified recombinant *T. cruzi* TryR for comparison [12]. Determinations for each enzyme were carried out in three independent experiments and a weighted mean calculated. T(S)_2_ concentration was varied from 5 × *K*_m_ to 0.5 × *K*_m_.

Substrate specificity in the 412 nm DTNB-coupled assay was investigated by comparing the reaction velocity in the standard assay mixture with reactions where 150 μM NADH was substituted for the NADPH and by comparing velocities of reactions containing 50 μM glutathione disulphide or 50 μM T(S)_2_. Values are presented as the mean and standard deviation of four replicates.

In order to determine *K*_m_ with respect to NADPH using the 412 nm DTNB assay, NADPH levels were maintained constant using glucose-6-phosphate and glucose-6-phosphate dehydrogenase from *Saccharomyces cerevisiae* [17]. Preliminary experiments over a range of NADPH concentrations (0.5–150 μM) established that 200 mU ml^−1^ of glucose-6-phosphate dehydrogenase was non-limiting (plateaux region of reaction velocity versus mU ml^−1^) and therefore chosen for determination of *K*_m_ with respect to NADPH. The final assay mixture contained 40 mM HEPES pH 7.4, 1 mM EDTA, ∼10 mU ml^−1^ TryR, 6 μM T(S)_2_, 50 μM DTNB and 0.2–200 μM NADPH (7-point 3-fold serial dilution). Assays were initiated by addition of the NADPH. *K*_m_ values with respect to NADPH were determined for *Tb*TryR in three independent experiments and a weighted mean calculated.

### 2.8 Enzyme inhibition studies

Inhibitor concentrations giving 50% inhibition (IC_50_) were determined using the 412 nm assay with the standard assay mixture modified to contain T(S)_2_ at [S] = *K*_m_. Assay plates were prepared using a Precision2000 liquid handler (Bio-Tek) with a final DMSO concentration of 1% in all wells. Plates contained a 10-point serial dilution (2-fold or 3-fold dilutions) across columns 2–11 with a top concentration of 100 μM or 200 μM. Seven compounds were tested per plate in rows A–G, row H was used for a clomipramine control on each plate. Column 1 contained full signal controls (no inhibitor) and column 12 contained background controls (no enzyme). All compounds were tested on three separate occasions and the results reported as weighted means. *Z*′ figures were calculated from the full signal and background controls [18].

### 2.9 Assessment of mode of inhibition

Clomipramine was tested for mode of inhibition with respect to T(S)_2_. The standard 412 nm DTNB-coupled assay was used with the following modifications. Aliquots of the assay mixture (180 μl) containing 0 μM, 1 μM, 2 μM and 4 μM clomipramine were added to four rows of a microtitre plate, respectively. T(S)_2_ was serially diluted across a fifth row of the plate to produce a 12-point range from 500 μM to 5.8 μM. The assay was initiated by transferring 20 μl of the T(S)_2_ row to each of the assay rows. The final 200 μl assay contained 150 μM NADPH, 50 μM DTNB, 10 mU ml^−1^ TryR and 50–0.58 μM T(S)_2_. The rate of reaction was measured as before. Each data set was fitted by non-linear regression to the Michaelis–Menten equation using GraFit 5.0 (Erithacus software). The resulting individual fits were examined as Lineweaver–Burke transformations and the graphs inspected to confirm competitive inhibition mode (intersection on *y*-axis). The entire data set was then globally fitted to the competitive-mode equation.

### 2.10 Growth inhibition studies

The effective concentration of compounds inhibiting cell growth by 50% (EC_50_) was determined as previously described [19,20]. Bloodstream form *T. b. brucei* cells (strain 427, ‘single marker’) were grown at 37 °C and 5% CO_2_ in a modified HMI9 [21] (HMI9-T where 0.2 mM 2-mercaptoethanol was replaced with 0.056 mM thioglycerol). Stock cultures were maintained in T75 vented cap culture flasks (Greiner, Kremsmuenster, Austria) and sub-cultured every 48–72 h by dilution into fresh medium. For microtitre plate assays, cells were counted using a Casy cell counter TT (Schärfe systems) and diluted appropriately. Compounds were tested in 96-well test plates (Greiner). The final conditions were 50–0.07 μM test compound (9-point 3-fold serial dilutions), 0.5% DMSO, 10^3^ ml^−1^ cells in a total volume of 0.2 ml. Plates were incubated for 3 days, resazurin was added to a final concentration of 45 μM and plates incubated for a further 4 h. Fluorescence was measured at 528 nm excitation and 590 nm emission. EC_50_ values were determined in three separate experiments and the data reported as weighted means.

### 2.11 Crystallography

*Tb*TryR was concentrated to 12.4 mg^−1^ ml in 20 mM bis-Tris buffer pH 7.0 containing 95 mM KCl, 1 mM EDTA and 1 mM DTT. The protein was crystallised by the hanging drop vapour-diffusion method at 20 °C using 24-well VDX plates with 500 μl of reservoir solution. Crystals were cryoprotected by sequential immersion for 5 s into a 3-μl drop containing 0.1 M bis-Tris propane pH 8.0, 5% PEG 400, 1 M ammonium sulphate plus 10% glycerol, then the same solution plus 20% glycerol and finally into another drop with 30% glycerol before flash-freezing them in liquid nitrogen. High resolution X-ray data to 2.3 Å was collected at with a MicroMax-007HF X-ray generator (Rigaku) and an R-axis IV detector (Rigaku) at a wavelength of 1.5428 Å. The X-ray data was integrated using MOSFLM [22], scaled with SCALA [23], and the structure solved using the *Tc*TryR structure [18,24], which shares 84% sequence similarity with *Tb*TryR, as the input model for molecular replacement with MOLREP [25]. Model building was carried out with COOT [26] and structure refinement with REFMAC5 [27,28].

## 3 Results and discussion

### 3.1 Cloning and expression

The gene encoding TryR was cloned from *T. b. brucei* strain 427 and found to be identical with that from the genome sequencing strain 927, apart from nucleotide substitutions of C for T at position 105 and A for G at position 906. Nevertheless, the two sequences are identical at the amino acid level. As noted above, TryR from *T. b. gambiense* is also identical at the amino acid level.

**Fig. 2:**
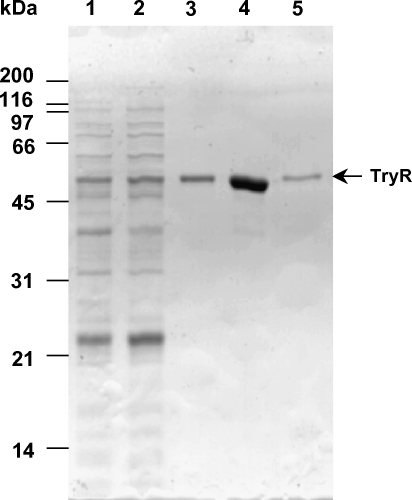
Analysis of trypanothione reductase purification by SDS-PAGE. Lane 1, crude cell lysate (2 μg); lane 2, protein after 35–70% ammonium sulphate cut (2 μg); lane 3, eluate from 2′5′-ADP Sepharose column (1 μg); lane 4, eluate from Q-Sepharose column (1 μg); lane 5, purified *T. cruzi* recombinant trypanothione reductase (0.2 μg).

*Tb*TryR was expressed in a 4 L culture of *E. coli* strain BL21 Star(DE3)pLysS competent cells and purified to apparent homogeneity (Fig. 2 and Table 1). The specific activity of the purified enzyme (91 U mg^−1^) is similar that of the *T. cruzi* (143 U mg^−1^) [12] and *L. donovani* (113 U mg^−1^) [29]. The overall yield of 7.6 mg l^−1^ is similar to the *T. cruzi* enzyme (19.0 mg l^−1^) [12] and *T. congolense* (3.2 mg l^−1^) [30]. Scaling up expression in a 30 L fermenter culture yielded 8.8 mg l^−1^ TbTryR.

### 3.2 Analysis of oligomeric state

The subunit mass of *Tb*TryR was calculated as 53,156 Da. Gel filtration revealed 86% of the sample eluting at a volume corresponding to 96 kDa (data not shown). Analytical ultracentrifugation indicated 90% of the sample was present at a molecular weight of approximately 93 kDa (data not shown). Both techniques therefore indicated the protein was almost entirely present as a dimer in solution.

### 3.3 Spectroscopic analysis

**Fig. 3:**
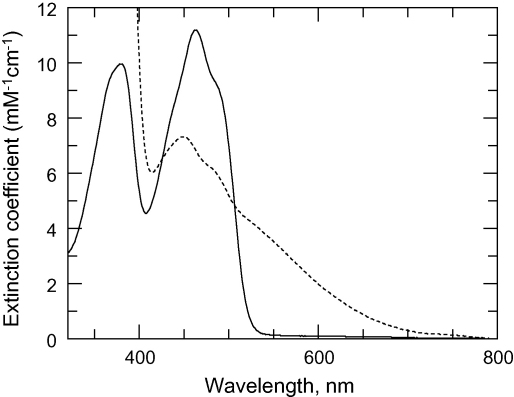
Absorption spectra of purified *TbTryR*. Solid line, oxidised enzyme; dashed line, 30 min after addition 2 mM NADPH.

Recombinant *Tb*TryR possesses spectral properties closely resembling those of other trypanothione reductases (Table 2 and Fig. 3) [31,32]. The oxidised enzyme (Fig. 3 solid line), where the redox-active cysteine residues, Cys52 and Cys57, within the disulphide binding site are covalently linked in a disulphide bridge, exhibits maxima at 273, 378 and 463 nm and a shoulder at 486 nm indicative of a flavoprotein [32]. Thermal liberation of the flavin prosthetic group yielded a mean absorption coefficient for the oxidised enzyme of 11.4 ± 0.3 mM^−1^ cm^−1^ at 463 nm (*n* = 4). Addition of excess NADPH (2 mM, Fig. 3 dotted line), leads to a decrease in the absorbance at 463 nm with concomitant acquisition of a broad long-wavelength absorption band at 530 nm due to reduction of the cysteine disulphide bridge and accompanying formation of a characteristic charge transfer complex between the FAD and the proximal sulphydryl group of Cys57 [29]. This spectrum is stable indefinitely in the presence of excess NADPH.

### 3.4 Substrate analysis

The *K*_m_ values for T(S)_2_ with saturating NADPH in both the 340 and 412 nm assays were compared with *T. cruzi* TryR (Table 2). The *K*_m_ values for the *T. cruzi* enzyme were 4.3- and 4.4-fold greater in each assay, respectively.

*Tb*TryR was highly specific for the electron donor (NADPH) and electron acceptor (T(S)_2_) in the 412 nm assay. Activity with 150 μM NADH was only 5.7% of that obtained with 150 μM NADPH and the rate of reduction of 50 μM glutathione disulphide was 0.03% of that obtained with 50 μM T(S)_2_.

We were unable to reliably determine a *K*_m_ with respect to NADPH in either the 340 nm direct assay or the 412 nm DTNB-coupled assay due to the short periods of linearity at low NADPH concentrations. Hence we developed a modified 412 nm DTNB-coupled assay wherein the NADPH level was maintained constant by inclusion of glucose-6-phosphate and glucose-6-phosphate dehydrogenase (Fig. 1C). The apparent *K*_m_ for NADPH determined in this modified assay was 0.77 μM.

The specificity constant (*k*_cat_/*K*_m_) for the *T. b. brucei* enzyme (8.7 × 10^6^ M^−1^ s^−1^) compares favourably with the previously reported values for *T. cruzi* (5.2 × 10^6^ M^−1^ ^−1^) [12,31] and *L. donovani* (5.0 × 10^6^ M^−1^ s^−1^) [29]. (Note that the lower value determined here for *T. cruzi* (2.6 × 10^6^ M^−1^ s^−1^; Table 2) is due to loss of ∼50% activity on prolonged storage at 4 °C as an ammonium sulphate suspension.)

### 3.5 Inhibitor testing

**Fig. 4:**
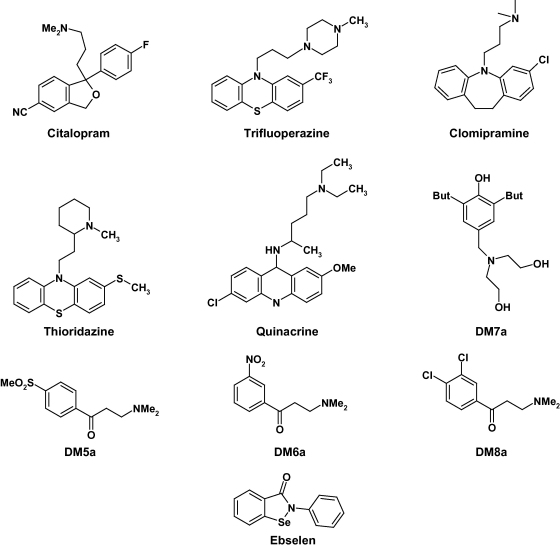
Structures of inhibitors tested against *Tb*TryR and *Tc*TryR. The following compounds were tested for potency of inhibition against *Tb*TryR and *Tc*TryR, see Table 3.

**Fig. 5:**
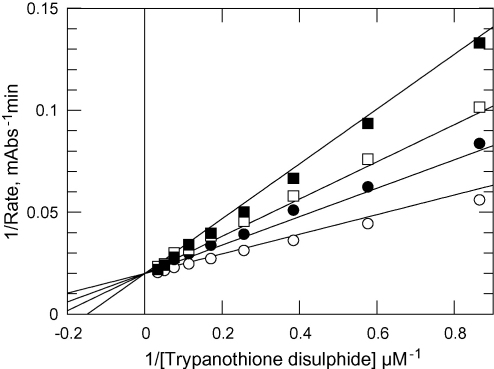
*K*_i_ determination of *Tb*TryR with respect to clomipramine. T(S)_2_ was varied as the substrate to confirm the mode of clomipramine inhibition and the *K*_i_ value. An *F*-test confirmed the mode of inhibition as linear competitive. Clomipramine was added at 0 μM (open circles); 1 μM (closed circles); 2 μM (open squares) and 4 μM (closed squares).

In order to identify potential differences in the sensitivity of trypanothione reductases from *T. b. brucei* and *T. cruzi* to small-molecule inhibitors, ten compounds spanning a range of inhibitor chemotypes, were tested for potency (IC_50_ values) against the two enzymes at T(S)_2_ concentrations equivalent to their respective *K*_m_ values. With these ten inhibitors the maximum difference between sensitivity of the two enzymes was 3.3-fold (Table 3 and Fig. 4). The mode of inhibition by clomipramine was confirmed as competitive for T(S)_2_ by measuring reaction velocity while varying T(S)_2_ at a range of clomipramine concentrations (Fig. 5). The *K*_i_ value from three independent experiments yielded a mean weighted to standard error of 3.03 ± 0.28 μM. This is similar to the calculated *K*_i_ value of 5.5 μM from our IC_50_ determination (for a competitive inhibitor at [S] = *K*_m_, IC_50_ = 2 × *K*_m_).

Ebselen, clomipramine and the four compounds DM5a; DM6a; DM7a and DM8a were known to be active against an *in vitro* assay of *T. b. brucei* proliferation [19,33]. Under identical conditions, the concentration inhibiting 50% growth (EC_50_) was determined here for three of the remaining TryR inhibitors, all of which were active in the single-figure micromolar range (Table 3). Despite the use of *Tb*TryR in place of *Tc*TryR used in previous studies [6], correlation between TryR inhibition and trypanocidal activity remains modest at best (*r*^2^ = 0.49). This indicates either significant differences in the uptake of these compounds or, more likely, that some of these compounds inhibit other targets within the cell.

Several of the compounds used in our study are currently in clinical use or have undergone human clinical trials; for example ebselen is being evaluated for the treatment of acute ischemic stroke [34]. Thioridazine, trifluoperazine and clomipramine have been previously identified as inhibitors of *Tc*TryR [35,36]. Although these CNS-active drugs show potent trypanocidal activity *in vitro*, thioridazine (50 mg kg^−1^, i.p.) and trifluoperazine (50 mg kg^−1^, i.p.) are inactive in the chronic mouse model of African trypanosomiasis [37] and clomipramine (50 mg kg^−1^, i.p.) failed to extend survival time by even one day in the acute mouse model (Halliburton and Fairlamb, unpublished). To our knowledge the structurally distinct antidepressant citalopram has not been tested *in vivo*. Despite the lack of *in vivo* activity, these molecules represent useful potential starting points for drug development for HAT, particularly as they are likely to cross the blood–brain barrier and therefore could be active against the late stage of the disease.

### 3.6 Crystallography

**Fig. 6:**
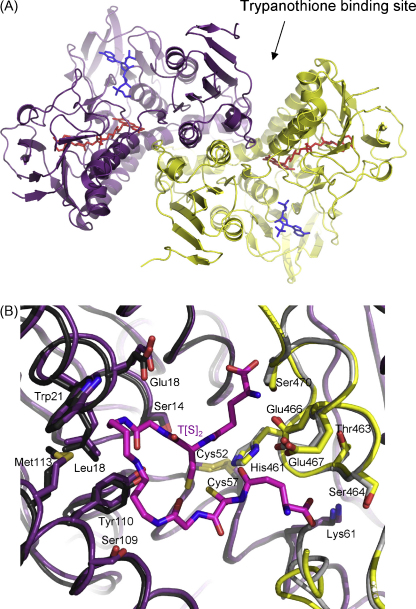
Crystal structure of *Tb*TryR. Panel A, the crystal structure is coloured to differentiate the two subunits forming the homodimer. FAD is shown in red and NADPH in blue. Panel B, a close-up of the *Tb*TryR active site (purple and yellow) superimposed onto the *Tc*TryR active site (black and grey, pdb code 1BZN ). Trypanothione is shown bound into the active site of the *Tc*TryR structure.

Large crystals (approximately 0.8 mm × 0.3 mm × 0.05 mm) diffracting to 2.3 Å were obtained in drops containing 2.0 μl of protein solution plus 1.0 μl reservoir solution, where the reservoir consisted of 0.1 M bis-Tris propane pH 8.0, 5% PEG 400, 2 M ammonium sulphate. Diffraction data was solved by molecular replacement using the *Tc*TryR structure; the overall statistics of the final *Tb*TryR model are given in Table 4. *Tb*TryR is a homodimer and each subunit of the final model comprises 489 amino acid residues, 1 FAD, 1 NADPH, 4 glycerol and 1 sulphate molecules. Also present are 786 solvent positions that were modelled as oxygen atoms and 1 PEG400 molecule (associated with subunit A). The first residue and the last 2 residues in both subunits were excluded from the structure as there was no convincing electron density to model. The same applies to the nicotinamide moieties of the two NADPH molecules, which were not included in the final model. The overall structure of *Tb*TryR (Fig. 6A) is nearly identical to that of *Tc*TryR (rmsd = 0.6 Å over 482 Cα atoms). All the catalytically important residues (Cys52, Cys57 and His461) and residues interacting with T(S)_2,_ including those involved in binding the spermidine moiety (Leu17, Glu18, Trp21, Met 113, Ser109, Tyr110) [24] are conserved between both structures (Fig. 6B).

## 4 Conclusion

In conclusion, the kinetic and physical properties of *Tb*TryR are consistent with trypanothione reductases from other trypanosomatids such as *T. cruzi* and *L. donovani*, especially the striking preference for T(S)_2_ over glutathione disulphide. We have observed no significant differences between the *Tb*TryR and *Tc*TryR in terms of *K*_m_, sensitivity to inhibitors or in crystal structure. Thus, either enzyme may be used as an effective surrogate for the other in high-throughput screening [6,19,33,38] and structure-based inhibitor design as part of a drug discovery campaign against human African trypanosomiasis or Chagas’ disease. One of Lipinski's “Rule of 5” [39] states that, for an orally bioavailable drug, the molecular mass should ideally be under 500 Da. Thus, one major challenge with druggability of this target is the large size of the active site, which must accommodate the substrates, T(S)_2_ (721 Da) or glutathionylspermidine disulphide (867 Da). In addition, a second challenge is the potential for displacement of reversible inhibitors from TryR by accumulation of T(S)_2_ as a consequence of further cellular metabolism of T[SH]_2_. Although we have now identified competitive inhibitors with *K*_i_ values ∼250 nM [33,40], theoretical calculations based on T[SH]_2_ concentrations in the cell suggest that *K*_i_ values ∼1–10 nM are required to sustain >90% inhibition in the face of accumulating T[S]_2_. Alternatively, irreversible active site directed inhibitors are required. Either strategy would be greatly enhanced with knowledge of the binding mode of inhibitors in the active site pocket of the enzyme. Details will be reported in subsequent publications.

## Figures and Tables

**Table 1:** Purification of recombinant *T. b. brucei* trypanothione reductase from *E. coli*. Activity was measured in the standard 340 nm assay as described in the methods.

Step	Volume (ml)	Total protein (mg)	Specific activity (U mg^−1^)	Total activity (U)	Purification factor *x*-fold	Yield (%)
Cell lysate	40	182	2.4	442	1	100
35–70% (NH_4_)_2_SO_4_	10.0	96.3	3.4	326	1.4	74
2′5′-ADP-Sepharose	17.8	4.5	78.4	353	32	80
Q-Sepharose	2.1	2.8	91.0	258	38	58

**Table 2:** Trypanothione reductase spectral properties and kinetic parameters. *K*_m_ values for T(S)_2_ were determined for *Tb*TryR and *Tc*TryR in both the 340 and 412 nm assays. Additional data from *L. donovani*[29] and *T. cruzi*[12,31].

Parameter	Units	*T. b. brucei*	*T. cruzi*	*L. donovani*
Spectral properties				
*λ*_max_ (oxidised enzyme)	nm	463	464	463
*ɛ*_0_ at *λ*_max_	mM^−1^ cm^−1^	11.4 ± 0.3	11.4	11.5
Charge transfer band (reduced enzyme)	–	Yes	Yes	Yes
*ɛ*_0_ at *λ*_530 nm_	mM cm^−1^	4.1	4.9	4.2

Enzymatic properties				
Specific activity	U mg^−1^	91	143	113
*K*_m_ NADPH (by DTNB-coupled assay)	μM	0.77 ± 0.01	n.d.^a^	n.d.^a^
*K*_m_ T(S)_2_ (by DTNB-coupled assay)	μM	2.35 ± 0.07	10.4 ± 0.3	n.d.^a^
*K*_m_ T(S)_2_ (by NADPH oxidation)	μM	6.9 ± 0.7	29.6 ± 2.8	36
*k*_cat_ (by NADPH oxidation)	s^−1^	46.8 ± 1.1	77 ± 8.0	179
*k*_cat_/*K*_m_	M^−1^ s^−1^	8.7 × 10^6^	2.6 × 10^6^	5 × 10^6^

a n.d.: not determined.

**Table 3:** Potency of representative compounds against trypanothione reductase from *T. b. brucei* and *T. cruzi* and growth inhibition of *T. b. brucei* cells*.* All IC_50_ values against *Tb*TryR and *Tc*TryR were determined in the 412 nm assay on three separate occasions. IC_50_ values were determined with T(S)_2_ at S = *K*_m_ (2.35 μM for *Tb*TryR and 10.4 μM for *Tc*TryR). For structures see Fig. 4. Linear regression analysis of IC_50_ for *Tb*TryR versus EC_50_ for *T. brucei* yielded a regression coefficient of *r*^2^ = 0.49.

Compound	IC_50_ (μM)		Ratio	EC_50_ (μM)
	*Tb*TryR	*Tc*TryR	*Tc/Tb*	*T. b. brucei*
Ebselen	0.18 ± 0.01	0.60 ± 0.01	3.3	2.97^a^
DM8a	3.17 ± 0.17	7.99 ± 0.51	2.5	1.00 ± 0.21^b^
DM6a	3.20 ± 0.16	3.80 ± 0.20	1.2	0.68 ± 0.10^b^
DM5a	6.80 ± 0.42	16.9 ± 1.08	2.5	0.55 ± 0.04^b^
Thioridazine	9.58 ± 0.38	10.0 ± 0.31	1.0	1.39 ± 0.03
Clomipramine	11.1 ± 0.42	3.41 ± 0.06	0.3	5.04^a^
DM7a	19.2 ± 1.75	27.8 ± 2.08	1.4	3.01 ± 0.3^b^
Quinacrine	22.1 ± 2.05	37.8 ± 1.91	1.7	n.d.
Trifluoperazine	40.5 ± 1.50	20.9 ± 0.90	0.5	2.11 ± 0.50
Citalopram	82.3 ± 6.35	154 ± 5.42	1.9	6.59 ± 0.54

a Data from Richardson et al. [33].

b Data from Martyn et al. [19].

**Table 4:** Crystallography statistics.

Space group	*P2*_*1*_*2*_*1*_*2*_*1*_
Cell dimensions: *a*, *b*, *c* (Å)	63.63, 132.71, 161.28
Cell angles: *α* = *β* = *γ* (°)	90
Molecules per asymmetric unit	2
Resolution (Å)	2.3
Measured reflections	292,320
Unique reflections	58,415
Completeness (%)	94.9 (85.7)^a^
Redundancy	5.0 (3.4)
*R*_sym_ (%)	0.049 (0.099)
〈*I*/*σ* (*I*)〉	22.9 (9.2)
Wilson *B* (Å^2^)	29
Overall *B*-factor (Å^2^)	21.0
*R*_free_ (5% of reflections)	0.227
*R*_factor_	0.181
Cruickshanks DPI	0.273

a The numbers in parentheses refer to the highest resolution bin (2.36–2.30 Å).
